# Scalable parallel ultrafast optical random bit generation based on a single chaotic microcomb

**DOI:** 10.1038/s41377-024-01411-7

**Published:** 2024-03-05

**Authors:** Pu Li, Qizhi Li, Wenye Tang, Weiqiang Wang, Wenfu Zhang, Brent E. Little, Sai Tek Chu, K. Alan Shore, Yuwen Qin, Yuncai Wang

**Affiliations:** 1 Institute of Advanced Photonics Technology, School of Information Engineering, Guangdong University of Technology, Guangzhou, 51006 China; 2 Key Laboratory of Photonic Technology for Integrated Sensing and Communication, Ministry of Education of China, Guangdong University of Technology, Guangzhou, 51006 China; 3 Guangdong Provincial Key Laboratory of Information Photonics Technology, Guangdong University of Technology, Guangzhou, 51006 China; 4 Key Laboratory of Advanced Transducers and Intelligent Control System, Ministry of Education, Taiyuan University of Technology, Taiyuan, 030024 China; 5 State Key Laboratory of Transient Optics and Photonics, Xi’an Institute of Optics and Precision Mechanics, Chinese Academy of Sciences, Xi’an, 710119 China; 6 Department of Physics and Materials Science, City University of Hong Kong, Hong Kong, China; 7 School of Electronic Engineering, Bangor University, Bangor, Wales LL57 1UT UK

**Keywords:** Optical techniques, Nonlinear optics

## Abstract

Random bit generators are critical for information security, cryptography, stochastic modeling, and simulations. Speed and scalability are key challenges faced by current physical random bit generation. Herein, we propose a massively parallel scheme for ultrafast random bit generation towards rates of order 100 terabit per second based on a single micro-ring resonator. A modulation-instability-driven chaotic comb in a micro-ring resonator enables the simultaneous generation of hundreds of independent and unbiased random bit streams. A proof-of-concept experiment demonstrates that using our method, random bit streams beyond 2 terabit per second can be successfully generated with only 7 comb lines. This bit rate can be easily enhanced by further increasing the number of comb lines used. Our approach provides a chip-scale solution to random bit generation for secure communication and high-performance computation, and offers superhigh speed and large scalability.

## Introduction

The security of digital networked society greatly depends on the extensive usage of randomness. In contrast to algorithmically generated pseudo-random bits, random bits extracted from physically stochastic processes, such as thermal noise and frequency jitter in electronic devices, can intrinsically ensure information security due to their unpredictability. Hence, physical random bit generators (RBGs), also referred to as true RBGs, are viewed as essential devices for guaranteeing the reliability of secure communication.

Shannon’s theorem establishes that it is required for the ultimate security to achieve bit rate matching that of the true RBGs with that of the communication systems^1^. For this purpose, optical chaos has been widely studied in the past decades as a means for the generation of broadband entropy sources to overcome the bit-rate bottleneck of traditional electronic RBGs^2–12^. However, because such chaotic sources usually produce just one channel of non-correlated stochastic intensity fluctuation, only one random bit stream can be generated for most available optical-chaos-based RBGs. Limited by the response speed of entropy extractors such as analog-to-digital converters (ADCs), it is very challenging for such sequential methods to continually improve their random bit generation rate so as to meet the ever-growing demands of advanced communication systems.

Parallel RBG schemes can solve this problem through multiplying the single-channel random bit rate by a number of non-correlated channels. Broad-area lasers subject to optical feedback^13^, cross coupled quarter-wavelength-shifted distributed feedback lasers^14^, cascaded phase-modulated semiconductor lasers^15^, and globally coupled semiconductor laser networks^16^ have been investigated for multi-channel chaos generation. Regrettably, the inter-channel correlation behaviors among chaotic fluctuations degrade the independence of parallel generated random bit streams. Moreover, their complicated coupling configurations limit their scalability.

Recently developed microcombs in optical micro-resonators (MRR) offer the possibility of photonic entropy sources for massively parallel random bit generation: a microcomb possesses hundreds of equally spaced comb spectral lines^17–24^. In particular, chaotic modulation instability (MI) combs exhibit temporal fluctuations in intensity^25–29^. This inspires us to envisage that hundreds of independent random bit streams may be simultaneously generated by spectrally demultiplexing chaotic micro-combs. However, there are two technical challenges hindering the use of such a promising entropy source for parallel RBGs: (i) The chaotic intensity fluctuation in each comb line always has an asymmetric amplitude distribution due to extreme events. This will introduce bias into the generated random bits; (ii) The chaotic microcomb always has a limited bandwidth of order 1 gigahertz (GHz). This will constrict the single-channel generation rate of random bits. Very recently, Shen et al. reported a fast parallel random bit generation using chaotic MRRs^30^. Through employing a special material AlGaAsOI (Aluminum Gallium Arsenide on insulator) based MRR, they enhanced the bandwidth of chaotic microcomb to several GHz and then realized a parallel RBG with a single-channel rate of 18 Gb/s. For enhanced performance, they need to photoelectrically mix two chaotic MRRs where an accurate frequency difference is carefully tuned to achieve broadband chaos. In any case, in using either one MRR or dual MRRs, there is a requirement for complicated post-processes including delay-difference and self-delayed multi-bit exclusive-OR in their method to eliminate the bias induced by the asymmetric distribution of the chaotic amplitudes. Such precise microcomb configurations and the associated complex post-processes militate against miniaturization and photonic integration which are essential to the practical deployment of such RBGs.

Herein, we present a RBG scheme using a single chaotic micro-comb, that not only can produce independent parallel random bit streams, but also can enhance the generation rate in a single channel. In addition to the ultrahigh speed, our method commends itself for its simplicity and scope for generalization. Without using any special materials, the chaotic microcomb in our experiment is produced by a CMOS-compatible, high-index, doped silica-glass MRR^31^. Through selecting the comb lines in designated areas, one can obtain parallel chaotic waveforms with symmetric distribution and no correlation. The detected chaotic waveforms are then over-sampled by their respective 16-bit ADCs and directly quantized into un-biased random bit streams only by retaining 8 least significant bits (LSBs). The use of over-sampling allows the extraction of ultrafast random bits from the bandwidth-limited chaotic comb, and also alleviates requirements on the front-end bandwidth of the ADCs.

A proof-of-principle experiment demonstrates that using this method, an ultrafast parallel physical RBG with a single chaotic microcomb can reach a 320 Gb/s generation rate in each channel, and a total bit rate of 2.24 terabit per second (Tb/s) can be obtained by only using 7 channels. Since the wavelength span of the chaotic microcomb is beyond 100 nm, our approach has the potential to provide hundreds of channels for parallel generation of independent random bit streams, and thus its cumulative rate can be further enhanced to order 100 Tb/s. More importantly, both the ultra-small size of the MRR and the simplified random bit extraction mean that this method is amenable to chip-scale parallel RBGs.

## Results

### Experimental configuration

**Fig. 1:**
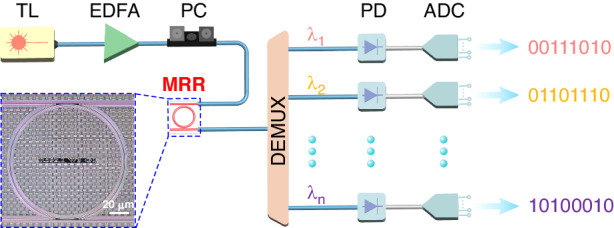
Experimental schematic for parallel random bit generation with a chaotic micro-comb. TL tunable laser, EDFA erbium-doped fiber amplifier, PC polarization controller, DEMUX optical demultiplexer, PD photodetecto, ADC 16-bit analog-to-digital converter, MRR micro-ring resonator. The dash box contains a microscope image of a high-index doped silica glass MRR with a radius of ~ 592.1 μm

Figure 1 illustrates the schematic of the parallel random bit generation using a single chaotic microcomb. The tunable laser (TL) is amplified by an erbium-doped fiber amplifier (EDFA), and then is coupled into the MRR as the pump light through a polarization controller (PC). The pump wavelength is adjusted to be very close to a resonance of the MRR from the blue side through controlling the operating temperature of the MRR by a temperature controller (TEC). As a result, a chaotic microcomb can be formed through carefully adjusting the pump power and the MRR operating temperature. Afterward, the obtained chaotic microcomb is spectrally split by a wavelength demultiplexer (DEMUX) into a series of chaotic waveforms with different central wavelengths from λ_1_ to λ_n_. These chaotic waveforms are converted into their respective electrical signals by their associated photodetectors (PDs). Finally, each chaotic signal is digitized into a binary stream by a 16-bit ADC for parallel random bit generation. A microscope image of the MRR used in this experiment is shown in the dashed box [Fig. 1]. The MRR is fabricated on a high-index doped silica glass (HIDSG) platform. An 8-μm-thick SiO_2_ layer is thermally oxidized on a silicon wafer as the lower cladding, while a 2-μm-thick HIDSG is deposited using a plasma enhanced chemical vapor deposition (PECVD) process. The waveguides are patterned using step lithography followed by reactive ion etching. The MRR has a radius of 592.1 μm with a waveguide cross-section of 2 × 3 μm, whose measured Q-factor is ~1.6 × 10^6^ at 1556.3 nm. It should be pointed that there is also no limit on the materials for MRRs. The other materials such as silicon nitride (SiN)^32^, silicon dioxide (SiO_2_)^33^ and lithium niobate (LiNbO_3_)^34^ should be also feasible for chaotic micro-combs. The main reason why we chose the HIDSG MRR in our experiment is its reliable packaging: this kind of MRR can be easily coupling using a fiber array with a low coupling loss. Well-packaged MRRs help to ensure reliable random bit generation and thus enable future applications.

### Flat Chaos in microcombs

**Fig. 2:**
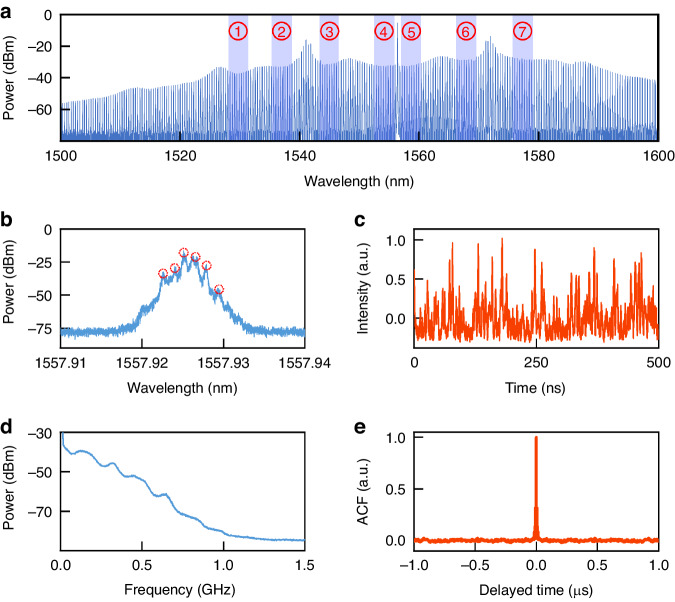
Characteristics of measured chaotic microcomb. **a** Optical spectrum of the chaotic microcomb, where the blue shaded areas indicate the comb lines used for parallel random bit generation. **b** Optical spectrum, **c** temporal waveform, **d** radio-frequency spectrum, and **e** autocorrelation function of a single comb-line in the blue shadow. Note, the red dashed circles represent the frequency components for flat chaos generation in Fig. 2b

Figure 2 illustrates the characteristics of the generated chaotic microcomb. The optical spectrum of the chaotic microcomb is measured using an optical spectrum analyzer with a resolution of 0.02 nm. The chaotic microcomb is generated by slowly decreasing the MRR operating temperature to sweep the MRR resonance from the blue-detuned regime to the pump wavelength. In our experiment, the wavelength of the pump laser is fixed at 1556.3 nm, while the initial operation temperature of the MRR is set to a relatively high temperature 47.8 °C. In this case, the pump wavelength can be located at the blue side of one MRR resonance. Afterward, we tune the temperature controller (TEC) so that the MRR resonance shifts towards the pump wavelength. With the reduce of the pump-resonance detuning, we can observe primary combs, sub-combs and chaotic combs in sequence. Figure 2 illustrates a typical chaotic microcomb when the MRR operating temperature drops to 36.6 °C. This microcomb exhibits an inherently spatial-temporal chaotic nature induced by MI, where approximately 250 comb lines can be observed within an ultra-wide spectral range from 1500 to 1600 nm. Note, there is no special requirement on the thermal stability for reliable random bit generation, because the chaotic microcomb is self-stable due to the negative feedback between the variation of the pump-resonance detuning and the thermal resonance shift^35^. In specific, the reduction of pump-resonance detuning will increase the intracavity power and thus cause an additional resonance shift towards a longer wavelength due to temperature increase. In turn, this red-shift results in the decrease of the intracavity power so that a resonance blue-shift of the MRR happens towards a lower wavelength. As a result, using a TEC controller with a precision of 0.1 °C is enough for stable chaotic microcomb surviving in our experiment.

In our approach, the comb lines in the blue shaded regions (labeled ①~⑦ in Fig. 2a) are used for parallel random bit generation. The main reason for choosing these regions is that they are located in the recesses of the spectral envelope, where each comb line has multiple frequency components. As a result of beating among these frequency components, a relatively flat radio-frequency (RF) spectrum can be obtained. Arbitrarily, we choose one of the comb lines from region ⑤ to illustrate the properties of the flat chaos as shown from Fig. 2b–e. Figure 2b is a typical fine spectral structure of one single comb line, which is measured by an optical spectrum analyzer with a high resolution of 0.04 pm. From the optical spectrum, we can confirm that there are at least six laser frequency peaks in the range of 1557.923 nm to 1557.931 nm, which are marked with red dashed circles in Fig. 2b. These frequency components beat with each other and modulate the optical field in the MRR, so that a broadband chaotic RF spectrum can be obtained as shown in Fig. 2d. It is to be expected that the RF spectrum is flat because the resonance peaks generated by the beat frequency are entirely independent. Figure 2c depicts the associated measured temporal chaotic waveform that exhibits large-amplitude oscillation and noise-like fluctuation. Its maximal Lyapunov exponent (MLE) is calculated to be 9.1372 using the method in ref. ^36^. A positive MLE means divergence and sensitivity to initial conditions and thus our system is considered to be in chaos. Furthermore, we obtain the autocorrelation function (ACF) of the measured chaotic waveform as plotted in Fig. 2e. Here, the time length of the used chaotic waveform is 25 µs. More details about the ACF see the section of Materials and methods: correlation analysis of chaotic waveforms. No correlation peaks are observed from the ACF. This δ-function-shaped characteristic guarantees high-quality generation of sequential random bits in each wavelength channel. In addition, we point out that the chaotic waveforms have a relatively symmetric distribution, as illustrated in Fig. 4a.

**Fig. 3:**
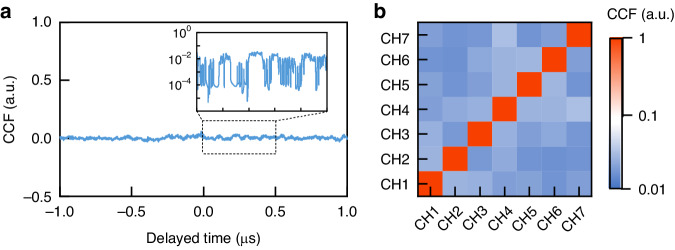
Cross-correlation characteristics of obtained parallel chaos. **a** Cross-correlation function between CH4 and CH5. **b** Cross-correlation function between any two channels shown as a confusion matrix

In addition to the chaotic waveform in each channel having no intra-channel correlation, a parallel RBG requires that there be no inter-channel correlation between chaotic waveforms in any two channels^37^. To simultaneously satisfy these criteria there is a need to select those comb lines whose central wavelengths are asymmetric about the pump wavelength, from the aforementioned blue shaded regions in Fig. 2a. In this proof-of-principle experiment, seven comb lines are taken as an example to establish the validity of this procedure. Specifically, the central wavelengths of these seven channels (named as CH1, CH2, CH3, CH4, CH5, CH6, CH7) are at 1530.66 nm, 1536.430 nm, 1544.564 nm, 1553.188 nm, 1557.940 nm, 1568.096 nm and 1577.596 nm, respectively. Figure 3a presents a typical cross-correlation function (CCF) between parallel chaotic temporal waveforms from any two different channels (such as CH4 and CH5), where the inset is a zoomed-in view from 0 to 0.5 μs. The CCF level below than 0.01 means that there is no significant correlation between the associated two chaotic waveforms. More CCFs are shown as a confusion matrix in Fig. 3b, where the color represents the CCF coefficients between any two channels. From it, one can further confirm that there is indeed no inter-channel correlation among the selected channels in accordance with the aforementioned criteria.

### Parallel random bit generation

Next, we use 16-bit ADCs to extract random bit streams from their respective chaotic waveforms as shown in Fig. 1. There are two keys for the generation of random bits with verified randomness: one is to improve the distribution non-uniformities of the output bits from the ADC; the other is to enhance the reduction of the auto-correlations among the output bits caused by the large sampling rate of the ADC. To achieve these two objectives, only 8-LSBs are retained to generate random bits in our method, albeit every sample point in the chaotic waveform is digitized into 16 bits by the ADC.

**Fig. 4:**
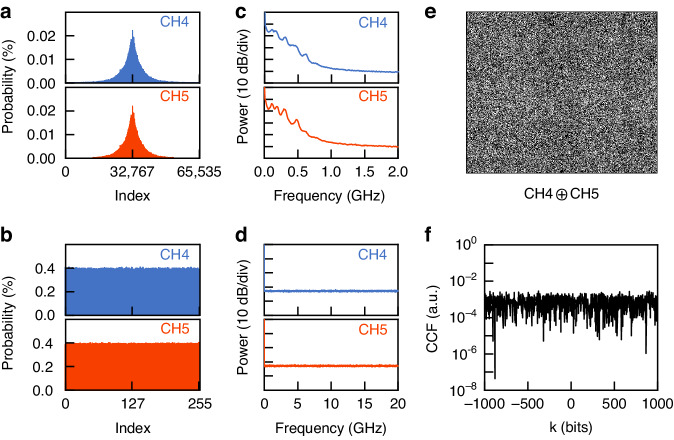
Comparison of extracted binary sequences from parallel chaos (CH4 and CH5). Amplitude histogram distributions (in integer representation) for the extracted binary sequences from CH4 and CH5 by retaining (**a**) 16-LSBs and (**b**) 8-LSBs; Power spectra of the extracted binary sequences from CH4 and CH5 by retaining (**c**) 16-LSBs and (**d**) 8-LSBs; (**e**) XOR bitmap image and (**f**) cross-correlation function (CCF) between the extracted binary sequences from CH4 and CH5 by retaining 8-LSBs

Figure 4 shows the associated properties of the random bit sequences generated from two adjacent channels (take CH4 and CH5 as examples). In this experiment, the chaotic waveform is oversampled by the ADC with a sampling rate of 40 GSa/s. Figure 4a shows the amplitude distributions of the binary bit sequences when all 16-LSBs are retained, respectively. The integers on the horizontal axis of Fig. 4a correspond to the decimal quantization values of the binary bit sequences. It is confirmed that for 16-LSBs, the chaotic amplitudes have a highly symmetric histogram due to the selection of comb lines in the designated areas identified by us. This feature allows the unbiased, even division of the bins based on the LSB of the bin as shown in Fig. 4b. However, when all 16-LSBs are retained, their bandwidth is very low as shown in Fig. 4c, which are Fourier transforms of the associated digitized signals in Fig. 4a. This limited bandwidth about 1 GHz is essentially inherited from the chaotic waveforms, and thus induces the correlation between the sampling points. To elimitate this intra-correlation in the binary bit sequences, we select only 8-LSBs as the final ranodm bit sequences. Figure 4d shows the power spectra of the digitized signals with 8-LSBs. Compared with all 16-LSBs [Fig. 4c], it can be seen that the bit truncation is equivalent to a spread spectrum operation: the digitized signals with 8-LSB show an ultrabroadband and nearly white spectrum. Subsequently, we construct 500 × 500 random bitmap images of the XOR results between CH4 and CH5 to evaluate the independence of the final random bit sequences. As shown in Fig. 4e, no obvious pattern, deviation, or correlation can be found. To further quantify the independence of the two random bit sequences, the cross-correlation functions (CCFs) are plotted in Fig. 4f. It is apparent that the inter-channel correlation coefficients are close to zero. That is, the generated parallel random bit streams are statistically independent.

**Fig. 5:**
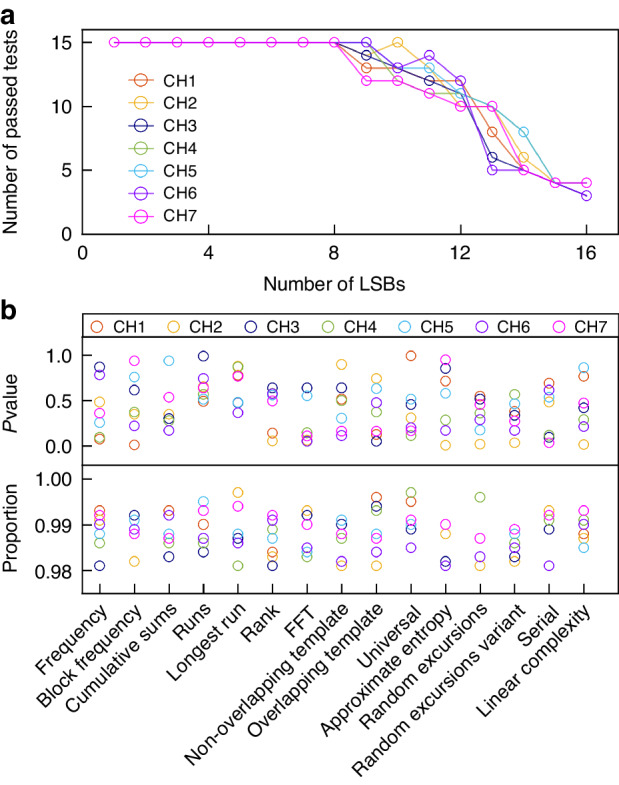
NIST test results. **a** Number of passed NIST tests as a function of number of LSBs for seven channels. **b** Results of NIST tests for parallel random bit streams from seven channels

Further, we use the standard statistical test suite of the NIST Special Publication 800-22 tests to more rigorously verify the statistical randomness of the generated parallel physical random bits. Figure 5 illustrates the associated test results. As suggested by the National Institute of Standards and Technology (NIST), we use 1000 instances of 1 million bits with a significance level of *α* = 0.01 for performing the NIST tests. Figure 5a shows the number of passed NIST tests when different LSBs are extracted as a random bit stream. The horizontal and vertical axes indicate the number of bits to retain LSBs in extracting random bits and the number of the passed NIST tests, respectively. It is seen that the random bit streams for the seven channels pass all of the NIST tests when the reserved bits of the LSBs ≤ 8. In addition, the detailed results of the NIST tests for the case of extracting 8-LSBs for each channel are also shown in Fig. 5b. From it, we can observe that all of the *P*-value are larger than 0.0001, and the probabilities of the tested random bits are higher than 0.9805608. Therefore, it is confirmed that the generated random bit streams for all seven channels are truly unpredictable in statistics. That is, we have successfully demonstrated the random bit stream with verified randomness of 2.24 Tb/s (7 × 40 × 8-LSBs) by extracting 8-LSBs at a sampling rate of 40 GS/s.

## Discussion

In summary, we have proposed and experimentally demonstrated a new approach to realize ultrafast parallel physical RBG based on a single chaotic microcomb. Compared to existing parallel RBG schemes, our method not only can greatly enhance the scalability and single-channel speed of parallel random bit generation, but also is extremely simple and highly efficient. In our proof-of-principle experiment, we successfully generated seven independent random bit streams with a single-channel speed of 320 Gb/s and an equivalent bit rate is as high as 2.24 Tb/s. This bit rate can be boosted to above 100 Tb/s through adding the channels. Taking advantage of the ultra-broadband spectrum and ultra-small size of the chaotic microcomb, our scheme provides an approach to ultrafast physical random bit generation which is amenable to on-chip implementation and thus is suitable for deployment in advanced communication systems.

In Table 1, we compare our work with some typical parallel physical random bit generators (RBGs) based on optical noise. For instance, Li et al. demonstrated simultaneous generation of two statistically independent 10 Gb/s random bit streams using amplified spontaneous emission from a single superluminescent LED^38^. Li et al. produced four independent 10 Gb/s random bit streams based on stochastic pulse-to-pulse fluctuation in a supercontinuum optical source^37^. Haylock et al. reported a parallel RBG using quantum vacuum state fluctuation in a laser system and seven random bit streams were successfully generated with a total bit rate of 3.08 Gb/s^39^. Different with optical noise, our proof-of-principle experiment demonstrates a 2.24 Tb/s (=320 Gb/s × 7) ultrafast parallel physical RBG with a single chaotic microcomb. More importantly, the ultra-small size of the micro-resonator ring enables our scheme to be a promising integrated physical entropy source.

**Table 1:** Comparison of our work with parallel physical RBG based on optical noise

Scheme	Channel	Bit rate	Reference
Amplified spontaneous emission	2	20 Gb/s	Li et al.^38^
Quantum vacuum state	7	3.08 Gb/s	Haylock et al.^39^
Optical supercontinuum	4	40 Gb/s	Li et al.^37^.
Optical microcomb	7	2.24 Tb/s	This work

There are at least two ways which can be used to further enhance the aggregated bit rate. (i) The less the Q factor, the higher the single-channel rate of the RBG. The single-channel rate of the RBG depends on the bandwidth of the flat chaos [Fig. 2d], which is essentially induced by the beating among the multiple frequency components in a single comb line [Fig. 2b]. When the linewidth of the comb line is broadened, more frequency components appear and thus the bandwidth of the flat chaos is enhanced correspondingly. As a result, the single-channel rate of the RBG can be further increased. Considering the Q factor is inversely proportional to the linewidth of the comb line^40^, we confirm that random bit streams with higher single-channel rates can be extracted from a chaotic micro-resonance ring (MRR) with a less Q factor. (ii) The narrower the channel (comb-line) spacing, the more the number of parallel channels of the RBG. On one hand, we can enhance the pump power to improve both the flatness (bandwidth) of the chaotic RF spectrum and the number of comb lines. On the other hand, the channel/comb-line spacing actually corresponds to the free spectral range (FSR) of the MRR. When the 3-dB micro-comb bandwidth is constant, the number of all comb-lines is inversely proportional to the FSR^41^. Therefore, we can design an MRR with a small FSR to further enhance the number of available channels for parallel random bit generation. Certainly, we can also optimize the waveguide dispersion of the MRR to broaden the microcomb spectrum bandwidth.

## Materials and methods

### Chaotic microcomb generation

The wavelength of the pump light source TL (EXFO T100S-HP) is 1556.3 nm with a linewidth of 400 kHz. After the EDFA (YOFC D-A-1550-5W), the output power of the TL is amplified into 5 W to pump the MRR. The operation temperature of the MRR is adjusted by the TEC (ILX Lightwave LDT-5412B) with a precision of 0.1 °C. When the operation temperature of the MRR is set to 36.6 °C, its resonance wavelength is very close to the pump wavelength of 1556.3 nm. The optical spectrum of the microcomb is recorded by the optical spectrum analyzer (YOKOGAWA AQ6370D) with a resolution of 0.02 nm and a scanning range from 1500 nm to 1600 nm. The comb lines are demultiplexed using several filters (EXFO XTM-50) with a bandwidth of 0.2 nm. The optical spectrum of each comb line is measured by the other optical spectrum analyzer (Apex AP2041B) with a high resolution of 0.04 pm. All the chaotic waveforms are detected by a 50 GHz PD (Finisar XPDV2120R) and recorded by a real-time oscilloscope (Lecroy LabMaster10-36Zi) with a bandwidth of 36 GHz and a sampling rate of 40 GS/s. Their associated RF spectra are measured by a spectrum analyzer (Rohde & Schwarz FSW50) with a resolution bandwidth of 10 MHz, a video bandwidth of 3 kHz and a measurement bandwidth of 50 GHz.

### Correlation analysis of chaotic waveforms

The ACF and the CCF of chaotic waveforms are calculated using the following formulas:

1 $${{ACF}}_{i}\left(\Delta t\right)=\frac{\left\langle \left({P}_{i}\left(t+\Delta t\right)-\left\langle {P}_{i}\left(t\right)\right\rangle \right)\left({P}_{i}\left(t\right)-\left\langle {P}_{i}\left(t\right)\right\rangle \right)\right\rangle }{{\left({\left\langle {P}_{i}\left(t\right)-\left\langle {P}_{i}\left(t\right)\right\rangle \right\rangle }^{2}{\left\langle {P}_{i}\left(t+\Delta t\right)-\left\langle {P}_{i}\left(t\right)\right\rangle \right\rangle }^{2}\right)}^{\frac{1}{2}}}$$

2 $${{CCF}}_{{ij}}\left(\Delta t\right)=\frac{\left\langle \left({P}_{i}\left(t+\Delta t\right)-\left\langle {P}_{i}\left(t+\Delta t\right)\right\rangle \right)\left({P}_{j}\left(t\right)-\left\langle {P}_{j}\left(t\right)\right\rangle \right)\right\rangle }{{\left({\left\langle {P}_{i}\left(t+\Delta t\right)-\left\langle {P}_{i}\left(t+\Delta t\right)\right\rangle \right\rangle }^{2}{\left\langle {P}_{j}\left(t\right)-\left\langle {P}_{j}\left(t\right)\right\rangle \right\rangle }^{2}\right)}^{\frac{1}{2}}}$$

where *P*_*i,j*_(*t*) represents the chaotic waveforms from the *i*-th and *j-*th channel, *∆t* is the time shift, and〈·〉denotes the time average. *ACF*_*i*_(∆*t*) indicates the autocorrelation function of the chaotic waveform in the *i*-th channel, while *CCF*_*ij*_(∆*t*) represents the cross-correlation function between the chaotic waveforms from the *i*-th and *j*-th channels.

### Random bit extraction and analysis

Random bit extraction is completed using an offline bit truncation. First, the chaotic waveform is sampled and recorded by the oscilloscope with an oversampling rate of 40 GS/s, which thus satisfy the Nyquist’s theorem for the chaos bandwidth about 1 GHz. Then, the 16-bit ADC divides the recorded chaotic data into 2^16^-1 bins, and each bin is coded into a binary sequence with a length of 16 bits (LSBs). Note, the comparator threshold corresponding to the 1-st MSB is set to the median of the chaotic data. At last, only 8 LSBs are retained to generate the random bit stream with verified randomness.

The CCF among the generated random bit streams is calculated as follows.

3 $${{CCF}}_{{ij}}\left(\Delta n\right)=\frac{\left\langle \left({x}_{i}\left(n+\Delta n\right)-\left\langle {x}_{i}\left(n+\Delta n\right)\right\rangle \right)\left({x}_{j}\left(n\right)-\left\langle {x}_{j}\left(n\right)\right\rangle \right)\right\rangle }{{\left({\left\langle {x}_{i}\left(n+\Delta n\right)-\left\langle {x}_{i}\left(n+\Delta n\right)\right\rangle \right\rangle }^{2}{\left\langle {x}_{j}\left(n\right)-\left\langle {x}_{j}\left(n\right)\right\rangle \right\rangle }^{2}\right)}^{\frac{1}{2}}}$$

where *x*_*i,j*_(*n*) represents the binary random bit streams generated from the *i*-th and *j*-th channel, respectively. *∆n* is the bit delay, while〈·〉denotes a statistical average of the binary random bit stream *x*_*i,j*_(*n*).

The random bitmap images are constructed from 250,000 binary random bits, which are obtained after XOR-processing the binary random bit streams in two different channels. The white and black dots in the bitmap are converted by the random bits “1” and “0” in the order from left to right and from top to bottom. For the amplitude histogram distributions, one must first convert the extracted binary sequences from different channels by retaining 16-LSBs or 8-LSBs into their respective decimal representations. Further, their associated power spectra are the Fourier transforms of the decimal representations. The NIST test suite (NIST SP 800-22) can be found from https://csrc.nist.gov/projects/random-bit-generation/documentation-and-software.

## Data Availability

The datasets generated and analyzed during the current study are available from the corresponding author upon reasonable request.
